# Use of Longitudinal EEG Measures in Estimating Language Development in Infants With and Without Familial Risk for Autism Spectrum Disorder

**DOI:** 10.1162/nol_a_00002

**Published:** 2020-03-01

**Authors:** Carol L. Wilkinson, Laurel J. Gabard-Durnam, Kush Kapur, Helen Tager-Flusberg, April R. Levin, Charles A. Nelson

**Affiliations:** Division of Developmental Medicine, Boston Children’s Hospital, Boston, MA; Division of Developmental Medicine, Boston Children’s Hospital, Boston, MA; Department of Neurology, Boston Children’s Hospital, Boston, MA; Department of Psychological and Brain Sciences, Boston University, Boston, MA; Department of Neurology, Boston Children’s Hospital, Boston, MA; Division of Developmental Medicine, Boston Children’s Hospital, Boston, MA

**Keywords:** autism spectrum disorder, electroencephalography (EEG), language development, biomarker, infant, child

## Abstract

Language development in children with autism spectrum disorder (ASD) varies greatly among affected individuals and is a strong predictor of later outcomes. Younger siblings of children with ASD have increased risk of ASD, but also language delay. Identifying neural markers of language outcomes in infant siblings could facilitate earlier intervention and improved outcomes. This study aimed to determine whether electroencephalography (EEG) measures from the first 2 years of life can explain heterogeneity in language development in children at low and high risk for ASD, and whether associations between EEG measures and language development are different depending on ASD risk status or later ASD diagnosis. In this prospective longitudinal study, EEG measures collected between 3 and 24 months were used in a multivariate linear regression model to estimate participants’ 24-month language development. Individual baseline longitudinal EEG measures included (1) the slope of EEG power across 3 to 12 months or 3 to 24 months of life for six canonical frequency bands, (2) the estimated EEG power at 6 months of age for the same frequency bands, and (3) terms representing the interaction between ASD risk status and EEG power measures. Modeled 24-month language scores using EEG data from either the first 2 years (Pearson *p* = 0.70, 95% CI [0.595, 0.783], *p* = 1 × 10^−18^) or the first year of life (Pearson *p* = 0.66, 95% CI [0.540, 0.761], *p* = 2.5 × 10^−14^) were highly correlated with observed scores. All models included significant interaction effects of risk on EEG measures, suggesting that EEG-language associations are different depending on risk status, and that different brain mechanisms affect language development in low- versus high-risk infants.

## INTRODUCTION

Children with autism spectrum disorder (ASD) have striking heterogeneity in their early language development (Anderson et al., 2007; Kjelgaard & Tager-Flusberg, 2001; Pickles, Anderson, & Lord, 2014). Although many children present initially with language delays, roughly one-fourth will develop age-appropriate skills by school age, and 30% will remain minimally verbal throughout life (Anderson et al., 2007; Tager-Flusberg & Kasari, 2013). Furthermore, language acquisition is one of the best predictors of later outcomes in children with ASD (Billstedt, Gillberg, & Gillberg, 2005; Gotham, Pickles, & Lord, 2012; Miller et al., 2017; Szatmari et al., 2000, 2009). Although children with ASD are clearly at risk for language delay, so are their younger siblings; for example, longitudinal studies following infant siblings of children with ASD (high-risk infants) have reported delays in language skills as early as 12 months of age in infant siblings, both with and *without* later diagnoses of ASD (Estes et al., 2015; Landa & Garrett-Mayer, 2006; Marrus et al., 2018; Swanson et al., 2017). As such, earlier identification of future language delays in toddlers at risk for ASD would facilitate earlier intervention and increase the likelihood of improving outcomes.

Longitudinal large population-based studies have identified various risk factors on the individual (gender, prematurity), familial (maternal education), and environmental (income) levels that influence language development (Reilly et al., 2007; Schjølberg, Eadie, Zachrisson, Oyen, & Prior, 2011). However, across studies these factors consistently account for 4%–7% of variance in language outcomes in 2-year-olds, suggesting that additional factors play an important role in a child’s early language trajectory. Indeed, high-risk infants who do *not* have ASD still have a fourfold increased risk of language delay compared to infants without a family history of ASD, suggesting that early language delay is an endophenotype of ASD with shared genetic liability and possibly shared underlying biology (Marrus et al., 2018). Therefore studying the neural mechanisms underlying language development in high-risk children may shed light on early language delay as a whole, and allow for earlier detection before delays in language can be measured behaviorally.

To meet this goal, researchers have applied neuroimaging techniques to measure brain-based changes that support language development in low- and high-risk infants over the first 3 years after birth. A number of studies suggest that the relationship between brain measures and language outcomes are different depending on ASD diagnosis or ASD risk status (Lombardo et al., 2015; Seery, Tager-Flusberg, & Nelson, 2014; Swanson et al., 2017). For example, in infants who show a language delay at a later age, the relationship between language ability and reactivity to speech (as measured on fMRI) is reversed depending on ASD diagnosis (Lombardo et al., 2015). Similarly, using EEG in low- and high-risk infants, significant relationships between frontal brain activity and language ability have been observed in high-risk but not low-risk infants at both 9 and 24 months (Seery et al., 2014; Wilkinson, Levin, Gabard-Durnam, Tager-Flusberg, & Nelson, 2019). These differential brain-behavior associations suggest that the neural mechanisms affecting language development in high-risk children may be different than in low-risk children. One reason for this could relate to global brain differences such as reduced power across frequency bands observed as early as 3 to 6 months in high-risk infants regardless of ASD outcome (Levin, Varcin, O’Leary, Tager-Flusberg, & Nelson, 2017; Riva et al., 2018; Tierney, Gabard-Durnam, Vogel-Farley, Tager-Flusberg, & Nelson, 2012), or accelerated surface area brain growth observed between 6 and 12 months of age in high-risk infants with later ASD diagnosis (Hazlett et al., 2017). These early brain differences could alter typical development of language circuitry. Given this, predictive models of language must account for these possible differences between low- and high-risk populations.

EEG, which measures network level brain activity at the scalp surface, has particular promise in its use as a clinical biomarker of language development in infants and toddlers as it is noninvasive, relatively low cost, and already regularly used in the outpatient setting for seizure monitoring (Jeste, Frohlich, & Loo, 2015; Levin & Nelson, 2015; Varcin & Nelson, 2016). Various measures collected during task-related or resting-state EEG have been associated with language processing and language development in young children. Evoked brain responses to auditory stimuli presented as early as 6 months of age reflect both current language processing and future language ability (Jansson-Verkasalo et al., 2010; Kuhl, 2010; Kuhl, Conboy, Padden, Nelson, & Pruitt, 2005). In addition, phase-locking, or entrainment, to both the temporal syllabic structure of speech (in the delta/theta range 1–8 Hz) and the phonetic structure of speech (in the beta/gamma range 20–50 Hz) has been observed in animals and humans during speech processing, and is correlated with measures of phonological processing (Giraud & Poeppel, 2012). Although it is still unknown whether aberrant entrainment in these frequency bands affects language acquisition in children, studies in children and adults with ASD have reported differences in resting and task-evoked theta and gamma power (Jochaut et al., 2015; Rojas & Wilson, 2014; Wang et al., 2013), and it is hypothesized that such differences impair speech processing and delay language acquisition (Jochaut et al., 2015). Clinically, resting EEG (non-task-related) is the simplest to collect, especially in young children or in populations for which longer task-related paradigms are not well tolerated. In addition, associations between resting frontal gamma power and language have been observed. Specifically, resting (i.e., not task-related) frontal gamma power has been associated positively with receptive and expressive language ability in typically developing 3- to 4-year-olds (Benasich, Gou, Choudhury, & Harris, 2008; Gou, Choudhury, & Benasich, 2011; Tarullo et al., 2017), but negatively associated with expressive language in high-risk 2-year-olds (Wilkinson et al., 2019). Reduced spectral power in delta/theta and gamma bands during visual processing has also been observed in minimally verbal children with ASD compared to a typically developing control group (Ortiz-Mantilla, Cantiani, Shafer, & Benasich, 2019). The spectral power of resting EEG has also been evaluated in children with dyslexia, with reduced frontal delta power observed in 3-year-olds who are later diagnosed with dyslexia compared with controls (Schiavone et al., 2014), but increased delta and theta power in school-aged children with dyslexia (Colon, Notermans, de Weerd, & Kap, 1979; Harmony et al., 1995; Penolazzi, Spironelli, & Angrilli, 2008; Spironelli, Penolazzi, Vio, & Angrilli, 2006), suggesting that developmental changes in EEG spectral power are also likely relevant predictors of language-related outcomes.

Although the preceding investigations have found associations between specific frequency bands and language development in both typically developing and neurodevelopmental disorder populations, they have not accounted for the majority of variance in language scores between individuals. This is likely because by focusing on a single frequency band during a limited developmental age range, we fail to capture the impact of developmental changes across the spectrum of frequency bands as it relates to language development.

This study had three main objectives. First, we aimed to evaluate whether a combination of early and longitudinal EEG measures (spectral power at 6 months of age and the developmental trajectory of spectral power) can explain the variance in language ability at 2 years of age in both low- and high-risk children. In other words, in a nonclinical longitudinal data sample, can resting EEG measures sufficiently estimate language ability in toddlers, to support its future use as a clinical biomarker of language development? Second, to begin to develop a theoretical framework of how neural oscillations (such as theta and gamma) may influence language development, we used a data-driven approach to model building, and then investigated whether associations between frequency band power and language ability were different between low- and high-risk infants. Specifically, in this second aim, we explored whether different EEG measures predict language ability in these risk groups, and whether the direction of these relationships was similar or different between groups. Because differences in EEG power have been observed as early as 3 to 6 months of age in high-risk infants, we hypothesized that spectral power at 6 months of age would differentially contribute to model language estimates in low- versus high-risk groups (LR and HR, respectively). In addition, we hypothesized that frequency bands important for speech processing (delta/theta and beta/gamma) would most robustly contribute to language estimates across low- and high-risk groups. Finally, we asked whether these brain-language associations were also different between high-risk infants with and without ASD (HR-ASD and HR-NoASD, respectively).

## METHODS

### Study Design and Population

Participants were part of a prospective longitudinal study of early neurocognitive development of infant siblings of children with ASD, conducted at Boston University and Boston Children’s Hospital. The study was approved by the institutional review board (#X06-08-0374) and written informed consent was obtained from a parent or guardian prior to each child’s participation in the study.

High-risk infants were defined as having at least one full sibling with a community diagnosis of ASD, which was confirmed using the Social Communication Questionnaire (Rutter Bailey & Lord, 2003) and/or the Autism Diagnostic Observation Schedule (ADOS; Lord & Rutter, 2012). Low-risk infants were defined by having a typically developing older sibling and no first- or second-degree family members with ASD. All infants had a minimum gestational age of 36 weeks, no history of prenatal or postnatal medical or neurological problems, and no known genetic disorders. For this analysis, all infants were also from households speaking primarily English (English spoken more than 75% of the time).

A total of 220 participants were enrolled who met the above inclusion and exclusion criteria (97LR, 123 HR). Only a portion of enrolled participants had sufficient quality EEG recorded (see EEG Rejection Criteria), developmental testing completed at the 24-month visit, and ADOS completed at 24 and/or 36 months. In addition, three low-risk male participants went on to meet Diagnostic and Statistical Manual of Mental Disorders, Fifth Edition (DSM-5) criteria for ASD and were not included in this analysis. This study includes data from a total of 58 LR and 72 HR infants (Table 1). Of the 72 HR infants, 21 (29%) met criteria for ASD based on assessments described in Language and Behavioral Assessments (Table 1).

**Table 1.** Sample characteristics

	LR N = 58	HR-NoASD N = 51	HR-ASD N = 21	*p* Value by outcome (LR vs HR-NoASD vs HR-ASD)	*p* Value by risk (LR vs HR^a^)
**Sex** ^b^	30M, 28F	25M, 26F	14M, 7F	0.4	0.86
**Parental education** ^b^, *n* (%)				0.060	0.04
Not answered	6 (10)	2 (4)	5 (24)		
<4-year college degree	3 (5)	10 (20)	3 (14)		
4-year college degree	10 (17)	10 (20)	6 (29)		
>4-year college degree	39 (67)	29 (57)	7 (33)		
**Household income** ^b^, *n* (%)				0.82	0.44
Not answered	8 (14)	3 (6)	6 (29)		
<$75,000	9 (15)	6 (12)	2 (9)		
>$75,000	41 (71)	42 (82)	13 (62)		
**Race** ^b^, *n* (%)				0.28	0.57
Non-white	7 (12)	3 (6)	4 (19)		
**Ethnicity** ^b^, *n* (%)				0.08	0.13
Hispanic or Latino	1 (2)	3 (6)	3 (14)		
**Number of EEG time points** ^c^ Mean ± SD	4.03 ± 1.1	3.64 ± 1.2	3.9 ± 1.3	0.23	0.13
**24m MSEL VDQ** ^d^ Mean ± SD	118.0 ± 15.3	112.0 ± 14.3	96 ± 22.6	0.0007	0.03
**24m MSEL NVDQ** ^d^ Mean ± SD	110.1 ± 15.0	107.0 ± 11.9	97 ± 22.6	0.02	0.14
**24m ADOS severity score** ^e^ Mean ± SD	1.61 ± 0.86	1.91 ± 1.13	5.25 ± 2.47	0.0001	0.003
**Participant EEG data included in analysis**, *n* (%)					
3 months	9 (15)	15 (29)	7 (33)		
6 months	44 (76)	30 (59)	13 (62)		
9 months	48 (83)	37 (73)	15 (71)		
12 months	52 (90)	33 (65)	19 (91)		
18 months	37 (64)	32 (62)	14 (67)		
24 months	44 (76)	39 (77)	14 (67)		

*Note*. ASD = autism spectrum disorder; LR = low-risk without ASD; HR-NoASD = high-risk without ASD; HR-ASD = high-risk with ASD; ADOS = Autism Diagnostic Observation Schedule.

^a^ HR = HR-NoASD + HR-ASD; *n* = 72.

^b^ Fisher exact.

^c^ One-way ANOVA.

^d^ One-way ANOVA with parental education as covariate.

^e^ Kruskal-Wallis.

### Measures

#### Language and behavioral assessments

At multiple time points, participants were assessed using the Mullen Scales of Early Learning (MSEL). For this analysis, the MSEL Verbal Developmental Quotient (VDQ) at 24 months was calculated from the receptive and expressive language subscales. This time point, instead of the later 36-month time point, was used as it included the most participants enrolled in the study. Final ASD outcomes were determined for all infants using the ADOS administered at 24 and 36 months of age. For those children meeting criteria on the ADOS, or coming within 3 points of cutoffs, a licensed clinical psychologist reviewed scores and video recordings of concurrent and previous behavioral assessments, and using DSM-5 criteria provided a best estimate clinical judgment.

#### EEG data collection

Baseline, non-task-related EEG data were collected at 6 visits (3, 6, 9, 12, 18, and 24 months of age). The infant was held by their seated caregiver in a dimly lit, sound-attenuated, electrically shielded room while a research assistant ensured that the infant remained calm and still by blowing bubbles and/or showing toys. Continuous EEG was recorded for 2 to 5 minutes. EEG data were collected using either a 64-channel Geodesic Sensor Net System or a 128-channel Hydrocel Geodesic Sensor Nets (Electrical Geodesics, Inc., Eugene, OR) connected to a DC-coupled amplifier (Net Amps 200 or Net Amps 300, Electrical Geodesics, Inc.). There was no difference in distribution of net type at each time point between outcome groups. Data were sampled at 250 Hz or 500 Hz, and collected using a 0.1-Hz high-pass analog (i.e., hardware) filter and referenced online to a single vertex electrode (Cz), with impedances kept below 100kΩ. Electrooculographic electrodes were removed to improve the child’s comfort.

#### EEG preprocessing

Raw EEG data collected in NetStation (Electrical Geodesics, Inc.) were exported to MATLAB (version R2017a) for preprocessing and subsequent power analysis.

All files were batch processed using the Batch EEG Automated Processing Platform (Levin, Méndez Leal, Gabard-Durnam, & O’Leary, 2018), to ensure uniform analysis regardless of when the EEG was acquired or which risk group they were in.

A 1-Hz high-pass filter and 100-Hz low-pass filter were applied and then data sampled at 500 Hz were resampled using interpolation to 250 Hz. Both experimental and participant-induced artifacts were then identified and removed using the Harvard Automated Preprocessing Pipeline for EEG (HAPPE; Gabard-Durnam, Mendez Leal, Wilkinson, & Levin, 2018). HAPPE is a MATLAB-based preprocessing pipeline optimized for developmental data with short recordings and/or high levels of artifact, to automate preprocessing and artifact removal, and to evaluate data quality in the processed EEG recordings (Gabard-Durnam et al., 2018). HAPPE artifact identification and removal includes removing 60-Hz line noise, bad channel rejection, and participant-produced artifact (eye blinks, movement, and muscle activity) through wavelet-enhanced independent component analysis (ICA) and multiple artifact rejection algorithm (MARA; Winkler, Debener, Muller, & Tangermann, 2015; Winkler, Haufe, & Tangermann, 2011). MARA has excellent detection and removal of muscle artifact components, which can affect higher frequency band signal (Gabard-Durnam et al., 2018; Winkler et al., 2011). The following channels, in addition to the 10–20 electrodes, were used for MARA: 64-channel net – 2, 3, 8, 9, 12, 16, 21, 25, 50, 53, 57, 58; and 128-channel net – 3, 4, 13, 19, 20, 23, 27, 28, 40, 41, 46, 47, 75, 98, 102, 103, 109, 112, 117, 118, 123. After artifact removal using HAPPE, data were re-referenced to an average reference (calculated using the same channels used for MARA), detrended using the signal mean, and then regions of high-amplitude signal (> 40 uV was used to account for the reduce signal amplitude which occurs during the wavelet-enhanced ICA step of HAPPE preprocessing) were removed prior to segmenting the remaining data into 2 s windows to allow for power calculations using multitaper spectral analysis (Babadi & Brown, 2014). Noncontinuous data were not concatenated.

#### EEG rejection criteria

HAPPE data output quality measures were used to systematically reject poor quality data that were unfit for further analyses. EEG recordings were rejected if they had fewer than 20 segments (40 s of total EEG), or were more than 3 standard deviations (SD) from the mean on the following HAPPE data quality output parameters: percent good channels (3 SD: < 82%), mean retained artifact probability (3 SD: > 0.3), median retained artifact probability (3 SD: > 0.35), percent of independent components rejected as artifact (3 SD: > 84%), and percent of EEG signal variance retained after artifact removal (3 SD: < 32%). Based on the preceding criteria, 69 of 674 (10.2%) of EEG recordings collected between 3 and 24 months were rejected. In addition, any EEG with a frequency band power greater than 2 SD above or below the mean of their associated outcome group (LR, HR-NoASD, or HR-ASD) mean were reviewed, resulting in the rejection of an additional 23 EEG studies (3.4%). HAPPE quality metrics and visual inspection rejection rates did not differ significantly between groups (all *p*’s > 0.1). A full description of HAPPE quality metrics and visualization based on this longitudinal dataset were published previously (Gabard-Durnam et al., 2018). Gabard-Durnam et al. (2018) also provide examples of EEG recordings from this dataset pre- and post-HAPPE processing, power spectra after each step of processing, and comparison of HAPPE versus other approaches to artifact rejection using this dataset.

#### EEG power analysis

A multitaper fast Fourier transform, using three orthogonal tapers (Thomson, 1982) was used to decompose EEG signal into power for each 2 s segment of data for selected frontal electrodes: 64-channel net – 2, 3, 8, 9, 12, 13, 58, 62; and 128-channel net – 3, 4, 11, 19, 20, 23, 24, 27, 118, 123, 124 (Supplemental Figure 1). Frontal electrodes chosen a priori based on findings from our previous work (Levin et al., 2017; Tierney et al., 2012; Wilkinson et al., 2019) and that of others (Benasich et al., 2008; Gou et al., 2011; Schiavone et al., 2014) showed that frontal power in both low- and high-frequency bands is associated with language development in typically developing and ASD populations. The summed power was calculated across all frequencies within commonly used frequency bands in infant EEG studies: delta (2–3.99 Hz), theta (4–5.99 Hz), low-alpha (6–8.99 Hz), high-alpha (9–12.99 Hz), beta (13–29.99 Hz), and gamma (30–50 Hz). As a minor point, we opted not to examine power < 2 Hz to allow use of a 1-Hz high-pass filter required by HAPPE. Power was then averaged across all 2 s segments and then across the frontal electrodes for each participant to obtain their average frontal power at each time point. We report absolute power values, normalized by a log 10 transform. Power spectra at each time point for low- and high-risk groups are shown in Supplemental Figure 2.

#### EEG data reduction

**Figure 1.**
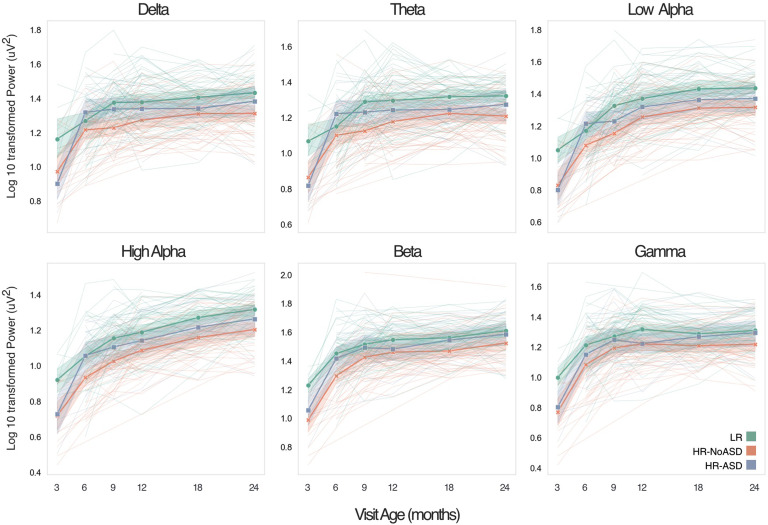
Developmental trajectories of frontal electroencephalography (EEG) power across multiple frequency bands from 3 to 24 months. Longitudinal trajectories of log 10 transformed absolute frontal EEG power across six frequency bands for individuals from low-risk (LR; green, *n* = 58), high-risk without ASD (HR-NoASD; orange, *n* = 51), and high-risk with ASD (HR-ASD; blue, *n* = 21) are shown. Both individual and mean trajectories by group are shown. Shaded regions represent the 95% confidence interval.

Longitudinal baseline EEG data from participants were reduced to two parameters for each frequency band per participant: (a) estimated power at 6 months (intercept at 6 months) and (b) estimated linear slope of power over logarithmic age, calculated either over 3 to 24 months (Models 1 and 2) or 3 to 12 months (Model 3). Given the nonlinear, logarithmic trajectories of EEG power over early brain development observed in this dataset (Figure 1) and by other labs (Cornelissen, Kim, Purdon, Brown, & Berde, 2015; Jing, Gilchrist, Badger, & Pivik, 2010), we employed the ordinary least squares to model a *linear* relationship between the log-transformed EEG power as a function of log-age for each individual. Ordinary least squares regression equations utilized all the EEG data available from each individual from 3 to 24 months. This linear transform allowed us to include longitudinal EEG data from any individual with EEG recordings from at least two time points across the 3- to 24-month period (Models 1 and 3) or 3- to 12-month period (Model 2), allowing us to maximize the sample size included in the model. The average number of EEG studies from each participant was 3.86 ± 1.2 and did not differ significantly between groups (Table 1; *p* > 0.1). The estimated 6-month intercept was used instead of a 3-month intercept because not all infants were enrolled at 3 months of age (the time of enrollment changed from 6 to 3 months during the study), and we aimed to use a time point that was inclusive of the full group’s study visit parameters.

### Statistical Analysis

#### Group comparisons of descriptive characteristics

A chi-square test (or Fisher exact test in case the cell value was < 5) was used to characterize differences in the categorical demographic data between groups. One-way analysis of variance (ANOVA) was used to assess group differences in average number of EEG studies per participant and MSEL scores. Kruskal-Wallis followed by post hoc Dunn’s test was performed on non-normally distributed ADOS severity scores.

### Model Development

Multivariate linear regression was used to characterize the relationship between the EEG parameters and 24-month language outcomes across groups. Three models were examined, all using 24-month VDQ as the dependent variable. Potential EEG predictors for each model included the 6-month intercepts, the slopes, and the interaction terms between intercept and slope for each of the six frequency bands (18 possible variables). For all adjusted models, parental education (averaged maternal and paternal education when both available), which is known to affect language development, was included as a covariate. Given the marginally significant increased number of male participants in the HR-ASD group, sex was also included as a covariate in adjusted models. Unadjusted models (Supplemental Materials) included only EEG parameters and interactions with either risk or ASD outcome.

For each of the models, we performed data-driven selection from the potential parameters using a hybrid stepwise approach and minimization of the Akaike information criterion (AIC) (Hastie, Tibshirani, & Friedman, 2009). This model selection uses an iterative process that compares AIC values across candidate models in order to minimize both information loss and overfitting. Once model selection was complete, secondary post hoc hypothesis testing was performed simultaneously on all parameters to determine which parameters were relatively robust predictors of MSEL VDQ and whether interaction effects were significant. Correction for multiple comparisons within the secondary analysis was performed using the Benjamini-Hochberg procedure (Benjamini & Hochberg, 1995) to limit the false discovery rate to alpha level 0.05 across tests, and *p* values surviving significance after correction are marked with an asterisk.

Model 1 included interactions between risk (LR vs. HR) and 15 of 18 EEG parameters calculated from data collected between 3 and 24 months. Model 2 included interactions between risk (LR vs. HR) and all 18 EEG parameters calculated from data collected from 3 to 12 months of age. Model 3, using data only from high-risk infants, included interactions between ASD outcome and 12 of 18 EEG parameters (Table 2). Due to the limited sample size, we could not simultaneously include interactions between EEG parameters and both risk and ASD outcome. In all models, B coefficients were scaled by a factor of 10, so that they represent the change in MSEL score after a 0.1-unit change in EEG intercept or slope.

**Table 2.** Predictive models of Mullen Verbal Developmental Quotient

**Model 1 (adjusted *R* ^2^ = 0.329)**						
	**Low risk**		**High risk**		**Difference (high – low risk)**	
	B Coefficient (SE)	*p* Value	B Coefficient (SE)	*p* Value	B Coefficient (SE)	*p* Value
**6-month intercept**						
Delta	3.55 (6.03)	0.56	20.07 (6.59)	**0.003** *	16.53 (8.15)	0.05
Theta	−7.04 (6.22)	0.26	−24.47 (6.69)	**<.001** *	−17.43 (8.26)	**0.04**
Low alpha	–	–	–	–	–	–
High alpha	7.92 (5.55)	0.15	4.51 (4.40)	0.31	−3.41 (6.44)	0.60
Beta	0.08 (4.66)	0.98	−0.1 (2.86)	0.97	−0.18 (4.68)	0.97
Gamma	−0.76 (2.37)	0.75	−0.76 (2.37)	0.75	−	−
**Slope**						
Delta	−2.91 (5.56)	0.60	−2.91 (5.56)	0.60	–	–
Theta	−13.50 (4.33)	**0.002** *	−13.50 (4.33)	**0.002** *	–	–
Low alpha	−0.21 (3.57)	0.95	5.41 (2.66)	**0.05**	5.61 (4.13)	0.18
High alpha	42.55 (16.68)	**0.01** *	8.97 (8.54)	0.3	−33.58 (18.02)	0.07
Beta	−77.88 (19.78)	**<0.001** *	−17.19 (9.35)	0.07	60.70 (20.29)	**0.004** *
Gamma	16.77 (6.18)	**0.008** *	16.77 (6.18)	**0.008** *	–	–
**Intercept × Slope**						
Delta	0.65 (0.32)	**0.04**	0.65 (0.32)	**0.04**	–	–
Theta	–	–	–	–	–	–
Low alpha	–	–	–	–	–	–
High alpha	−3.28 (1.48)	**0.03**	−0.04 (0.87)	0.97	3.24 (1.69)	0.06
Beta	5.33 (1.24)	**<0.001** *	0.81 (0.72)	0.26	−4.53 (1.42)	**0.002** *
Gamma	−1.33 (0.51)	**0.01** *	−1.33 (0.51)	**0.01**	–	–
**Covariates**						
Sex, reference = female	−6.96 (2.93)	**0.02**	−6.96 (2.93)	**0.02**	–	–
**Parental education**						
College degree	13.05 (4.91)	**<0.01** *	13.05 (4.91)	**<0.01** *	–	–
>College degree	16.94 (4.37)	**<0.001** *	16.94 (4.37)	**<0.001** *	–	–

**Model 2 (adjusted *R* ^2^ = 0.249)**						
	**Low risk**		**High risk**		**Difference (high – low risk)**	
	B Coefficient (SE)	*p* Value	B Coefficient (SE)	*p* Value	B Coefficient (SE)	*p* Value
**6-month intercept**						
Delta	−1.16 (5.48)	0.83	11.17 (6.19)	0.08	12.33 (8.04)	0.13
Theta	7.25 (7.33)	0.33	−16.07 (6.14)	**0.01**	−23.32 (9.44)	**0.02**
Low alpha	−2.41 (3.86)	0.54	1.76 (3.44)	0.61	4.16 (5.24)	0.43
High alpha	–	–	–	–	–	–
Beta	−4.28 (4.08)	0.30	2.52 (2.86)	0.38	6.80 (4.28)	0.12
Gamma	5.45 (3.46)	0.12	0.61 (2.87)	0.83	−4.84 (3.84)	0.21
**Slope**						
Delta	–	–	–	–	–	–
Theta	1.05 (1.93)	0.59	−2.75 (1.78)	0.13	−3.80 (2.57)	0.14
Low alpha	8.21 (5.01)	0.11	−3.06 (2.82)	0.28	−11.27 (5.82)	0.06
High alpha	–	–	–	–	–	–
Beta	−19.09 (7.39)	**0.01**	−1.39 (7.65)	0.86	17.70 (8.79)	**0.048**
Gamma	6.11 (4.09)	0.14	6.11 (4.09)	0.14	–	–
**Intercept × Slope**						
Delta	–	–	–	–	–	–
Theta	–	–	–	–	–	–
Low alpha	−0.70 (0.44)	0.12	0.47 (0.21)	**0.03**	1.17 (0.50)	**0.02**
High alpha	–	–	–	–	–	–
Beta	1.41 (0.49)	**0.005**	0.22 (0.56)	0.69	−1.18 (0.62)	0.06
Gamma	−0.51 (0.32)	0.12	−0.51 (0.32)	0.12	–	–
**Covariates**						
Sex, reference = female	−7.14 (3.11)	**0.02**	−7.14 (3.11)	**0.02**	–	–
**Parental Education**						
College degree	5.73 (4.93)	0.24	5.73 (4.93)	0.24	–	–
>College degree	13.40 (4.55)	**0.004**	13.40 (4.55)	**0.004**	–	–

**Model 3 (adjusted *R* ^2^ = 0.530)**						
	**No autism**		**Autism**		**Difference (autism − no autism)**	
	B Coefficient (SE)	*p* Value	B Coefficient (SE)	*p* Value	B Coefficient (SE)	*p* Value
**6-month intercept**						
Delta	12.49 (5.78)	**0.04**	34.59 (14.37)	**0.02**	22.10 (15.53)	0.16
Theta	−15.95 (6.42)	**0.02**	−53.11 (18.94)	**0.008** *	−37.16 (20.02)	0.07
Low alpha	−3.23 (3.91)	0.41	7.12 (7.34)	0.34	10.35 (7.97)	0.20
High alpha	9.08 (4.58)	0.06	9.08 (4.58)	0.06	–	–
Beta	−7.13 (3.48)	**0.047**	20.73 (7.81)	**0.01**	27.86 (8.63)	**0.003** *
Gamma	7.66 (3.74)	**0.048**	−15.56 (6.19)	**0.02**	−23.22 (7.35)	**0.003** *
**Slope**						
Delta	–	–	–	–	–	–
Theta	−2.89 (4.34)	0.51	−23.17 (11.93)	0.06	−20.29 (12.29)	0.11
Low alpha	−13.04 (5.42)	**0.02**	4.97 (11.10)	0.66	18.01 (10.92)	0.11
High alpha	9.19 (4.89)	0.07	9.19 (4.89)	0.07	–	–
Beta	12.39 (10.88)	0.26	28.84 (12.81)	**0.03**	16.46 (9.02)	0.08
Gamma	5.26 (3.39)	0.13	−4.63 (6.19)	0.46	−9.89 (7.07)	0.17
**Intercept × Slope**						
Delta	–	–	–	–	–	–
Theta	–	–	–	–	–	–
Low alpha	1.30 (0.40)	**0.002** *	1.30 (0.40)	**0.002** *	–	–
High alpha	–	–	–	–	–	–
Beta	−1.61 (0.77)	**0.04**	−1.61 (0.77)	**0.04**	–	–
Gamma	–	–	–	–	–	–
**Covariates**						
Sex, reference = female	−6.50 (3.62)	0.08	−6.50 (3.62)	0.08	–	–
**Parental Education**						
College degree	9.25 (5.44)	0.09	9.25 (5.44)	0.09	–	–
>College degree	11.80 (4.64)	**0.015**	11.80 (4.64)	**0.015**	–	–

*Note*. B Coefficient are scaled by a factor of 10. For example, B Coefficient for the 6-month intercept represents change in Mullen Scales of Early Learning Verbal Development Quotient for every 0.1uV increase in spectral power. *p* Values in bold are significant. SE = Standard error.

* Maintained significance after false discovery rate (FDR) correction, using an FDR at alpha = 0.05.

## RESULTS

### Participant Characteristics

Demographic data are shown in Table 1 for the full sample, with statistical analyses comparing both outcome groups (LR vs. HR-NoASD vs. HR-ASD) and risk groups (LR vs. HR, with HR consisting of combined HR-NoASD and HR-ASD infants).

### Developmental EEG Trajectories

Developmental trajectories of EEG power across the six frequency bands are shown in Figure 1, subdivided by outcome group (LR, HR-NoASD, and HR-ASD).

#### Longitudinal EEG measures explain language variability in low- versus high-risk infants (Model 1)

**Figure 2.**
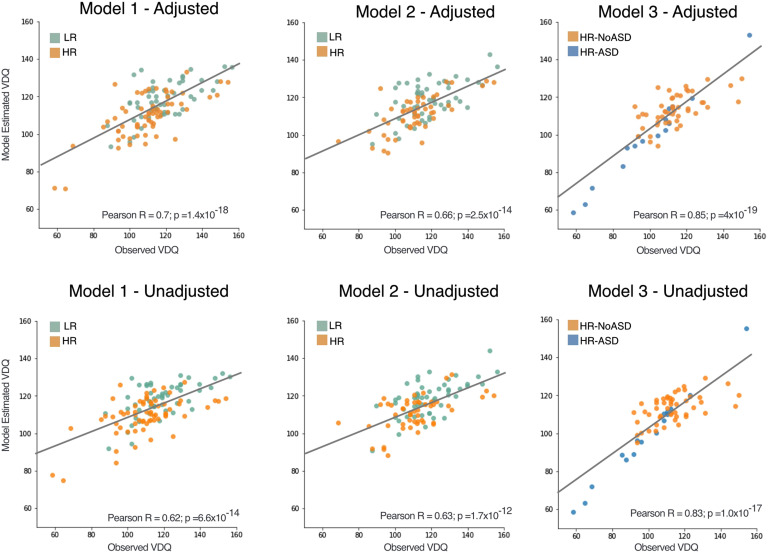
Correlations of observed and model estimated verbal developmental quotient (VDQ). Observed and estimated Mullen Scales for Early Learning VDQ for each model. Adjusted models include sex and parental education as covariates. Regression line shown represents estimate based on all individuals included in the model. Each data point is colored based on the independent variable included in the model (Models 1 and 2 = risk status; Model 3 = ASD diagnosis).

To test whether EEG predictors of language were different in low- versus high-risk infants, Model 1 allowed for potential two-way interactions between risk status and all EEG parameters included in the model. Pearson correlations between the modeled language scores and observed language scores were all significant, both collapsed across low- and high-risk subjects (Model Adjusted *R* ^2^ = 0.329; Pearson *R* = 0.70, 95% CI [0.595, 0.783]; *p* = 1 × 10^−18^) and when low-risk (Pearson *R* = 0.617, 95% CI [0.414, 0.762]; *p* = 1 × 10^−6^) and high-risk (Pearson *R* = 0.709, 95% CI [0.542, 0.812]; *p* = 4 × 10^−11^) groups were analyzed separately. Pearson correlation for the unadjusted model (Model Adjusted *R* ^2^ = 0.211), using only EEG parameters, was also significant (Pearson *R* = 0.62, 95% CI [0.497, 0.722]; *p* = 6.6 × 10^−14^). Scatterplots of model-estimated and observed language scores are shown in Figure 2.

Significant differences between risk groups were observed for the effects of beta slope and its interaction with its 6-month intercept as well as the effect of 6-month theta intercept on language outcomes (Table 2, Model 1). Four EEG parameters were significantly associated with language outcome in both low- and high-risk groups and had the same B coefficient: theta slope, gamma slope, delta intercept × slope, and gamma intercept × slope. Notably, risk itself was not a significant predictor of 24-month language in either the adjusted or unadjusted models (Adjusted Model 1: B coefficient = 19.31, SE 52.22; *p* = 0.71; Unadjusted Model 1: B coefficient = −33.9, SE 58.64; *p* = 0.56). Parental education and sex covariates were significant predictors in the adjusted model (Table 2, Model 1). The unadjusted Model 1 had similar patterns of significance (data shown in Supplemental Table 1).

#### Estimating 2-year language from EEG measures over the first year of life (Model 2)

We next examined how well the longitudinal EEG measures restricted to visits from the first year of life predicted language ability at 24 months across low- and high-risk infants. Model 2 used EEG data from both low- and high-risk infants, but now restricted to data collected between 3 and 12 months. Pearson correlations between estimated and actual MSEL scores were significant (Model Adjusted *R* ^2^ = 0.249; Pearson *R* = 0.66, 95% CI [0.540, 0.761]; *p* = 2.5 × 10^−14^; Figure 2), although not as accurate as Model 1. As in Model 1, beta slope and its interaction with the beta 6-month intercept were significantly associated with future language ability *only* for low-risk infants, and 6-month theta intercept was associated with future language ability *only* for high-risk infants; however, neither remained statistically significant after controlling for multiple comparisons. Gamma slope and the interaction between intercept and slope were no longer significant predictors in this model, suggesting that the trajectory of gamma power beyond 12 months may be more relevant to language development than its trajectory over the first 12 months.

#### Differences in how EEG measures explain language variability in high-risk infants with and without ASD (Model 3)

Next we asked whether high-risk infants with ASD (HR-ASD) have different brain-language associations than high-risk infants without ASD (HR-NoASD). Model 3 therefore used EEG data only from high-risk infants, allowing for two-way interactions between ASD outcome and EEG parameters. This model had improved predictive accuracy as measured by the correlation between model-estimated and measured language outcomes (Figure 2) across high-risk infants (Adjusted Model: Adjusted *R* ^2^ = 0.530; Pearson *R* = 0.849, 95% CI [0.763, 0.905]; *i* = 4 × 10^−19^; Unadjusted Model: Adjusted *R* ^2^ = 0.480; Pearson *R* = 0.83, 95% CI [0.737, 0.894; *p* = 1 × 10^−17^). As expected, ASD diagnosis negatively contributed to language outcomes, but was only marginally significant as a predictor (B coefficient = −123.0, *p* = 0.053). In this adjusted model, two brain-language associations were significantly different between high-risk infants with and without ASD: beta and gamma 6-month intercepts. For each of these, the brain-language association was significant within each group, but directionally opposite (Table 2, Model 3). Higher estimated 6-month gamma power was associated with increased 24-month language in HR-NoASD infants, and decreased 24-month language in HR-ASD infants.

In addition, several longitudinal EEG measures contributed similarly across both groups. Consistent with Model 1, both HR-NoASD and HR-ASD groups had significant and consistent associations between 6-month intercepts of theta and delta and 24-month language measures. In addition, the effect of the interaction between intercept and slope for the low alpha and beta frequency bands were the same across groups.

## DISCUSSION

This work represents our effort to utilize longitudinal EEG measurements to predict a functionally relevant developmental outcome in ASD—language ability. In addition, we were able to further dissect differences in brain-language associations between low- and high-risk infants crucial to furthering our understanding of the neural underpinnings of language delay in this population.

### Use of EEG as a Predictor of Language Ability

All three models were remarkably accurate in within-model estimates of language ability. Of interest, unadjusted models (which did not include parental education and sex covariates) also showed a high correlation between model-estimated and observed language scores, emphasizing the importance of the brain measures in the predictive models. In addition, in our adjusted Model 1, ASD risk status was not a significant predictor of language ability. This suggests that when the model accounts for differences in brain-language associations related to risk status, as well as sex and parental education, the remaining effect of high-risk ASD status on language development is no longer significant. In addition, significant interactions between several EEG variables and risk status were present in the model, supporting our hypothesis that there are significant brain differences between low- and high-risk infants, and that these brain differences rather than risk status contribute to language development.

Although EEG parameters in this study sample could estimate language scores with reasonable accuracy, there are several major caveats that need to be addressed before we are fully convinced that EEG measures can be used clinically to accurately predict future language risk. First, relatively few participants in this study sample had below average language skills. To improve generalizability, a much larger sample with diverse language ability is required. Second, the ability of EEG to be used as a clinical biomarker is reliant on EEG measures being reproducible across different locations and different collection environments. Third, although we used resting power for this study, other measures such as intertrial phase coherence, phase amplitude coupling, or newly developed measures such as fitting oscillations & one over f, or FOOOF (Haller et al., 2018), should also be evaluated to determine which measures best discriminate language outcomes. Finally, external validity of model performance needs to be tested in the future.

#### Further insights into neural mechanisms of early language development

A secondary goal of this study was to evaluate differences in brain-language associations between low- and high-risk infants. To do this we performed post hoc analyses of the predictors included in the models to determine which EEG measures most robustly contributed to language estimates and whether there were significant differences between low- and high-risk infants in how a measure contributed to language estimates. In our models, possible EEG measures included both a developmentally early measure of EEG power (estimated 6-month intercept) and a measure of how EEG power changes over development (slope as a function of log-age). We specifically included estimated 6-month power, as we have previously reported reduced EEG power across frequency bands at this young age in the high-risk population (Levin et al., 2017; Tierney et al., 2012) and hypothesized that this reduced power may influence the development of language circuitry. Indeed, in Models 1 and 2, early estimated 6-month EEG power measures (in delta and theta) contributed significantly to language scores *only* in the high-risk group. One possible explanation for this is that these early power measures reflect the degree of aberrant brain development or differences in cortical maturation in this at-risk population. Consistent with this, in our model restricted only to high-risk infants (Model 3), estimated 6-month power of multiple frequency bands (delta, theta, beta, and gamma) played prominent roles in predicting language.

We also hypothesized that specific frequency bands known to be important during speech processing (delta/theta and beta/gamma) would be significant predictors of language within our models; however, we were uncertain whether brain-language associations in these frequency bands would be different between risk groups, as differences in resting state and task-evoked theta and gamma power have been observed in children and adults with ASD. However, in evaluating specific frequency bands it is important to recognize that within the multivariate linear regression models developed, the contribution of an individual variable exists in the context of the other variables included (EEG measures, risk status, sex, parental education). Therefore, the effect attributed to a particular variable reflects only the *unique* portion of that variable that does not overlap with the other variables included in the model. Thus, interpreting the direction and weight of B coefficients within these models must be done with caution, as the effects of any frequency band may be different in the multivariate model context than when the same frequency band is examined in isolation. With this caveat in mind, several observations related to theta and gamma warrant further discussion.

Within Model 1, which included data from 3 to 24 months from low- and high-risk infants, the slope of theta and gamma were significant predictors of language and did not differ between risk groups. In addition, estimated 6-month delta and theta power were also significant predictors of language in this model, but only for the high-risk group. In particular, estimated 6-month theta power was a consistent significant predictor in all three models for high-risk infants, both adjusted and unadjusted, with increased theta power associated with worse 24-month language ability. Notably, increased theta power has been associated with learning and attention disorders (Barry, Clarke, & Johnstone, 2003), and increased theta has been observed in institutionalized infants and toddlers with increased risk for developmental delays (Marshall, Bar-Haim, & Fox, 2002). There are several biological explanations for theta’s association with language outcomes. Perhaps most intriguing is the observation that during speech processing, neural oscillations in the delta and theta range phase-lock to the syllabic rhythm of speech, which typically peaks in the theta range (Giraud & Poeppel, 2012; Power, Mead, Barnes, & Goswami, 2012). Aberrant phase-locking in the delta and theta range has been proposed as a possible mechanism for abnormal language acquisition (Goswami, 2011). In addition, theta oscillations are known to influence gamma activity and this coupling between frequencies is thought to facilitate the alignment of speech components during speech decoding (Giraud & Poeppel, 2012). In adults with ASD, atypical coordination between theta and gamma responses to speech has been reported, and correlated with clinical measures of ASD (Jochaut et al., 2015). Future investigation of theta oscillations and their coupling with gamma oscillations, at rest and in response to speech, at this young age in neurodevelopmental populations at risk for significant language delays may provide specific insight related to auditory processing abnormalities affecting language development. Because theta oscillations have also been implicated in attention, future comparison of models developed to estimate other developmental nonverbal skills (e.g., visual-spatial, motor, attention) would provide more information of the specificity of these EEG patterns on language development.

Alterations in theta and gamma power have been observed in children and adults with ASD who also have language delays; therefore it is possible that the association between these frequency bands and language within Model 1 may be related to their shared variance with ASD. To further separate out EEG measures that predict language, beyond their association with ASD, we developed Model 3, which includes ASD diagnosis as a predictor and interaction term with EEG measures. Here, having an ASD diagnosis was a marginally significant independent predictor of language (*p* = 0.05) with a large negative B coefficient, suggesting that factors associated with ASD, but not captured by other variables in our model, have a negative impact on language development. Such factors could include other structural and functional brain-derived measures besides resting EEG power, or reduced eye contact or aberrant sensory processing that may not be captured by resting EEG measures. In addition, with the ASD variable in the model, *shared* variance between ASD status and the EEG measures is no longer reflected in the individual parameters but does contribute to the model’s overall ability to differentiate language scores. It is notable that within Model 3, significant and opposing differences in brain-language associations of estimated 6-month beta and gamma power were observed when ASD status was included in the model. Here we observe that when ASD diagnosis and its effect on language outcomes are accounted for, reduced 6-month gamma power positively contributes to estimated 24-month language ability. However, when the effects of ASD on language are not included in the model, reduced 6-month gamma power negatively contributes to estimated 24-month language ability.

Gamma oscillations are associated with many processes thought to be relevant to language processing, including visual and auditory sensory integration (Senkowski, Schneider, Foxe, & Engel, 2008), attention (Fries, Nikolić, & Singer, 2007; Taylor, Mandon, Freiwald, & Kreiter, 2005), and working memory (Howard et al., 2003; Pesaran, Pezaris, Sahani, Mitra, & Andersen, 2002). In infants, induced gamma-band power in response to native phonemes matures between 3 and 6 months of age (Ortiz-Mantilla, Hämäläinen, Musacchia, & Benasich, 2013; Peña, Pittaluga, & Mehler, 2010). Resting frontal gamma power has also been positively associated with language ability in toddlers and preschool-aged children (Benasich et al., 2008; Brito, Fifer, Myers, Elliott, & Noble, 2016; Gou et al., 2011; Tarullo et al., 2017). However, increased gamma power has been observed in older children with ASD and other ASD-related neurodevelopmental disorders such as Fragile X syndrome, and this aberrant increase in gamma power is thought to reflect imbalances in the excitatory and inhibitory systems of the brain (Buzsáki & Wang, 2012; Sohal, 2012; Traub et al., 2003; Whittington & Traub, 2003). Furthermore, in Fragile X syndrome, increased baseline gamma power has been associated with decreased stimulus-induced phase-locked gamma activity, and therefore has been hypothesized to represent an hyperexcitable system with reduced ability to synchronize to a stimulus (Ethridge et al., 2016). In minimally verbal children with ASD, reduced frontal gamma during visual processing has been observed and hypothesized to be modulated by attention, although how this related to baseline gamma has not been reported (Ortiz-Mantilla et al., 2019). Thus, one hypothesis for the difference between gamma-language associations in HR-NoASD versus HR-ASD infants, is that reduced gamma power in an individual with underlying excitatory/inhibitory imbalance (HR-ASD) may represent successful compensation of early aberrant neurocircuitry, whereas reduced gamma power in an individual without such underlying imbalances (HR-NoASD) may reflect delayed neural maturation as it relates to speech processing. Future investigation of how differences in resting gamma may influence evoked gamma across development may inform both the timing and development of therapeutics.

Finally, sex and parental education both were significantly associated with language in adjusted models, supporting an independent role of each in language development. This is not surprising as previous studies have observed relationships between both sex (Campbell et al., 2003; Choudhury & Benasich, 2003; Reilly et al., 2007; Schjølberg et al., 2011; Zubrick, Taylor, Rice, & Slegers, 2007) and parental education (Campbell et al., 2003; Schjølberg et al., 2011; Tomblin, Hardy, & Hein, 1991; Tomblin, Smith, & Zhang, 1997) and language development. However, unadjusted models that used EEG data alone also estimated language scores that were highly correlated with actual scores, suggesting that longitudinal EEG measures may also reflect differences in sex and parental education. Of interest, in Model 3, which was restricted to high-risk infants and included ASD outcome, sex continued to have a marginally significant effect on language outcomes. Given the growing evidence of sex differences in early brain development within ASD (Baron-Cohen, 2010; Kim et al., 2013; Lai et al., 2017; Mottron et al., 2015; Werling & Geschwind, 2013), it is possible that within the high-risk infant-sibling population, there are additional brain-language differences between males and females. A larger sample size is needed to fully tease apart the effects of sex and parental education on brain development as it relates to language development in this complex population.

### Limitations

Although this is a comparatively large infant-sibling study, we were limited in our analyses by our sample size. To maximize the number of individuals included in model development, we included individuals with only two EEG time points (n = 21). Requiring more time points could provide more stable estimations of trajectories; however, it would also significantly reduce the sample size. Indeed, limiting Model 1 to individuals with at least three EEG sessions, reduced model fit (adjusted *R* ^2^ = 0.2574 vs. 0.3295) and accuracy (Pearson correlation coefficient 0.65 vs. 0.70). This study also limited its analysis to frontal power. The decision to use frontal power was made a priori based on previous resting-state EEG literature in both the ASD and language fields. However, it is possible that power analysis using a different set of electrodes could be more informative. To evaluate this possibility, models using whole brain or temporal electrodes were built from the same individuals included in Model 1. Both of these models had reduced adjusted *R* ^2^ values (whole brain, 0.2065; temporal, 0.2334; frontal, 0.3295) and lower Pearson correlation coefficients for predicted versus observed Mullen scores (temporal, 0.59; whole brain – 0.60, frontal – 0.70). Finally, as discussed earlier, in order to improve generalizability of future predictive models, a larger and more diverse study sample is required, followed by external validity testing of model performance.

### Conclusions

This study supports the possible use of EEG measures as predictive biomarkers for language development in infants. It also provides further insight into which neurobiological substrates may specifically relate to language development, potentially informing future work and therapeutic interventions. In addition, our findings support further investigation of brain differences in high-risk infants at, or prior to, 6 months of age, and how such differences affect future language development and response to services. To do this, collaboration across laboratories is needed in order to collect larger datasets for accurate model development that can then be leveraged clinically. Ultimately, early prediction of developmental outcomes will require improved knowledge of how underlying genetic and environmental risk factors affect neural measures *and* their association with outcomes.

## ACKNOWLEDGMENTS

We thank all the families and staff who were involved in this study.

## FUNDING INFORMATION

H. Tager-Flusberg, National Institute on Deafness and Other Communication Disorders (https://doi.org/10.13039/100000055), Award ID: R01-DC010290. C. A. Nelson, National Institute on Deafness and Other Communication Disorders (https://doi.org/10.13039/100000055), Award ID: R01-DC010290. H. Tager-Flusberg, National Institute on Deafness and Other Communication Disorders (https://doi.org/10.13039/100000055), Award ID: R21 DC08637. C. L. Wilkinson, National Institute of Mental Health (https://doi.org/10.13039/100000025), Award ID: 1T32MH112510. A. R. Levin, American Brain Foundation (US). C. L. Wilkinson, Autism Science Foundation (https://doi.org/10.13039/100008152). L. J. Gabard-Durnam, Autism Science Foundation (https://doi.org/10.13039/100008152). A. R. Levin, Autism Science Foundation (https://doi.org/10.13039/100008152). H. Tager-Flusberg, Autism Speaks (https://doi.org/10.13039/100000073), Award ID: 1323. A. R. Levin, Brain and Behavior Research Foundation (https://doi.org/10.13039/100000874). C. L. Wilkinson, FRAXA Research Foundation (https://doi.org/10.13039/100000297). A. R. Levin, N. Lurie Marks Family Foundation (https://doi.org/10.13039/100007429). C. L. Wilkinson, Thrasher Research Fund (https://doi.org/10.13039/100005627). L. J. Gabard-Durnam, Rett Syndrome Research Trust. Charles A Nelson, Simons Foundation (https://doi.org/10.13039/100000893), Award ID: 137186.

## AUTHOR CONTRIBUTIONS

C. L. Wilkinson: Conceptualization – lead; Data curation – lead; Formal analysis; Methodology; Writing – original draft. L. J. Gabard-Durnam: Conceptualization; Methodology; Software; Writing – review & editing. K. Kapur: Formal analysis; Methodology – lead; Writing – review & editing. H. Tager-Flusberg: Conceptualization; Funding acquisition; Investigation; Supervision; Writing – review & editing. A. R. Levin: Methodology; Software; Supervision; Writing – review & editing. C. A. Nelson: Conceptualization; Funding acquisition; Investigation; Project administration – lead; Supervision; Writing – review & editing.
